# Protective Efficacy of a Chimeric *Pestivirus* KD26_E2LOM Vaccine Against Classical Swine Fever Virus Infection of Pigs

**DOI:** 10.3390/v17040529

**Published:** 2025-04-05

**Authors:** Young-Hyeon Lee, Bo-Kyoung Jung, Song-Yi Kim, Dohyun Kim, Min-Kyung Jang, SeEun Choe, Byung-Hyun An, Jae-Jo Kim, Yun Sang Cho, Dong-Jun An

**Affiliations:** 1 Virus Disease Division, Animal and Plant Quarantine Agency, Gimcheon 39660, Gyeongbuk-do, Republic of Korea; yhlee916@korea.kr (Y.-H.L.); jbok429@korea.kr (B.-K.J.); songkim@korea.kr (S.-Y.K.); doh936@korea.kr (D.K.); mkjang0506@korea.kr (M.-K.J.); ivvi59@korea.kr (S.C.); jkim1209@korea.kr (J.-J.K.); choys@korea.kr (Y.S.C.); 2 Department of Virology, College of Veterinary Medicine and Research Institute for Veterinary Science, Seoul National University, Seoul 08826, Republic of Korea; anbh5043@gmail.com

**Keywords:** BVDV, CSFV, chimeric, pig, pestivirus, vaccine

## Abstract

A chimeric *pestivirus* KD26_E2LOM strain can induce antibodies that can be partially distinguished from antibodies from classical swine fever virus (CSFV) infection. The chimeric *pestivirus* vaccine strain was created using bovine viral diarrhea virus as the backbone; however, the entire BVDV E2 gene region was replaced with the E2 gene, which encodes the major target for neutralizing antibodies against CSFV. Pigs were vaccinated once or twice with the chimeric *pestivirus* KD26_E2LOM strain, and protective efficacy was evaluated after subsequent challenge with virulent CSFV. Pigs inoculated with the chimeric *pestivirus* KD26_E2LOM strain did not have a high temperature or leukopenia, and CSFV neutralizing antibodies (>64-fold) were observed from 28 days postvaccination (dpv). In addition, the level of anti-CSFV E2 antibody positivity was >0.8 (s/*p* value) from 30 dpv, and there were no antibody-positive individuals among the sentinel pigs. In control pigs, CSF antigen was detected in blood, nasal, and fecal samples at 5, 7, 10, 14, and 21 days postchallenge (dpc) and in several organs; however, no CSFV was detected in the organs of pigs vaccinated with the chimeric *pestivirus* KD26_E2LOM strain, and no virus shedding or CSF antigen was detected on any dpc. Thus, the chimeric *pestivirus* KD26_E2LOM strain protects pigs against horizontal transmission of virulent CSFV; however, this strain may have only partial potential for the differential detection of CSFV E^rns^ antibodies.

## 1. Introduction

Classical swine fever (CSF), an endemic disease in many pig-producing countries worldwide, has high mortality rates, which, coupled with international trade restrictions, result in enormous economic losses [1,2]. Classical swine fever virus (CSFV; genus *Pestivirus*; family *Flaviviridae*) carries a single-stranded positive-sense RNA genome of approximately 12.3 kb in length, with an open reading frame (ORF) encoding four structural proteins (C, E^rns^, E1, and E2) and eight non-structural proteins (N^pro^, p7, NS2, NS3, NS4A, NS4B, NS5A, and NS5B) [3,4]. Modified live vaccines (MLVs) based on existing attenuated CSFV strains can be very powerful tools for controlling CSF [2,5], but they do not allow naturally infected animals to be distinguished from vaccinated animals; therefore, research into DIVA (differentiating infected from vaccinated animals)-based live CSFV marker vaccines is underway.

Chimeric marker vaccines, used in combination with DIVA (differential detection) testing strategies that distinguish infected from vaccinated animals, have been approved for many economically important animal pathogens, including *Varicellovirus bovinealpha1* (synonym: bovine herpesvirus type 1 [BoHV-1]), *Varicellovirus suidalpha1* (synonyms: Aujeszky’s disease virus [ADV] or pseudorabies virus [PRV]), and *Pestivirus suis* (synonym: classical swine fever virus [CSFV]) [6,7,8]. The recent discovery that *pestiviruses* are very distant relatives of CSFV has provided new options for designing improved CSF marker vaccine candidates based on the chimeric *pestivirus* concept; this strategy has proven to be one of the most promising for generating efficient MLVs [9]. The recent development of CSFV marker vaccine candidates as well as solving the problem posed by chimeric *pestiviruses* closely related to CSFV has led to the design of chimeric pestiviruses that are genetically and antigenically distant from CSFV using a chimeric construction method that replaces the viral glycoprotein E^rns^ [10]. As a result, three chimeric viruses, “Ra”, “Pro”, and “RaPro”, were generated by replacing the E^rns^ sequence of the CSFV Alfort–Tübingen strain with those of Norway rat *pestivirus* (NRPV) or vegetative *pestivirus* (PAPeV) or a combination of the two [10]. In addition, a Japanese group recently reported creation of two chimeric viruses, vGPE^−^/PAPeVE^rns^ and vGPE^−^/PhoPeVE^rns^, that provide rapid and efficient protection against systemic infection by CSFV of intermediate virulence; this was achieved by combining a live attenuated CSFV vaccine strain with the glycoprotein E^rns^ from a distant relative of CSFV [11,12]. Recently, we constructed a chimeric *pestivirus* (called KD26_E2LOM) by inserting the E2 gene of the CSFV LOM strain into the backbone of the bovine viral diarrhea virus (BVDV) KD26 strain [13]. The safety and immunogenicity of the KD26_E2LOM strain, even at very high doses, were confirmed in growing pigs, pregnant sows, and calves [13]. KD26_E2LOM was shown to be safe in animals, but its ability to prevent infection by virulent CSFV infection was not evaluated.

Therefore, the purpose of this study was to examine the ability of the KD26_E2LOM strain to protect pigs against challenge with CSFV. Pigs were vaccinated once or twice, and the potential of the vaccine as a first-line vaccine as well as its ability to protect against horizontal transmission of CSFV from pig to pig were assessed. In addition, the potential for serological differentiation (DIVA), which is an advantage of the chimeric virus (KD26_E2LOM strain), was investigated.

## 2. Materials and Methods

### 2.1. Efficacy of the KD26_E2 LOM Strain in Animals

To determine the ability of the KD26_E2 LOM strain to protect pigs against challenge with virulent CSFV, 60-day-old pigs (CSF antigen and antibody negative) were inoculated with the KD26_E2 LOM vaccine. The SW03 strain (genotype 2), a moderately virulent CSFV isolated from Korean pigs in 2003 [14], was administered intramuscularly as the challenge strain. This experiment also investigated the difference of CSFV E^rns^ antibodies in distinguishing infection in vaccinated animals between pigs vaccinated once and twice with KD26_E2 LOM vaccine.

In total, 18 pigs were used for the experiment. The pigs were divided into five groups: group 1 (n = 5) was inoculated once with the KD26_E2 LOM vaccine; group 2 (n = 2) was used as sentinels for group 1; group 3 (n = 5) was inoculated twice with the KD26_E2 LOM vaccine; group 4 (n = 2) was used as the sentinels for group 3; and group 5 (n = 4) was used as a positive control group. The first and second vaccinations with the KD26_E2 LOM vaccine were administered intramuscularly at a dose of 10^4.0^ TCID_50_/mL (IM)/dose, with an interval of 3 weeks between the two doses. In addition, the pigs in groups 2 and 4 were housed together with those in groups 1 and 3, respectively, from the time of KD26_E2 LOM vaccination. Samples (blood, nasal, and feces) were collected from pigs in each group at 0, 3, 5, 7, 10, 14, 21, 24, and 28 days after KD26_E2LOM vaccination (dpv), and additional samples were collected from pigs in groups 3, 4, and 5 at 30, 33, and 35 dpv. Pigs in group 1, vaccinated with the KD26_E2 LOM vaccine, were challenged with the SW03 strain (10^6.0^ TCID_50_/mL/dose) at 28 dpv, and those in group 3 were challenged at 35 dpv. Pigs in group 5 (the positive control group) were also challenged with the SW03 strain (but not vaccinated). Pigs in groups 2 and 4 were housed together with the pigs in groups 1 and 3, respectively and used as sentinel animals (these animals were not inoculated with virulent CSFV). To ensure that the CSFV challenge dates were simultaneous, pigs in group 1 were vaccinated with KD26_E2LOM 1 week later than pigs in group 3. Body temperature was measured daily for 21 days postchallenge (dpc) with SW03, and samples (blood, nasal, and feces) were collected at 0, 2, 5, 7, 10, 14, and 21 dpc.

### 2.2. Real-Time PCR (qRT-PCR) to Detect CSFV and BVDV

Viral RNA was extracted from blood, fecal, and nasal samples using the RNeasy Mini kit (Qiagen Inc., Cat. No. 74104, Valencia, CA, USA) and then used for qRT-PCR to measure the CSFV RNA copy number. This qRT-PCR analysis was performed using the HelixDtec CSF Detection & Typing kit (Nanohelix Co., Cat. No. CSF-T100, Daejeon, Republic of Korea), which is designed to detect the 5′-UTR and E^rns^ of CSFV. This kit differentiates between virulent CSFV and the CSF vaccine strains (LOM strain and Flc-LOM-BE^rns^ strain) used in South Korea. The qRT-PCR was performed on a CFX Opus 96 Real-Time PCR System (Bio-Rad Laboratories, Inc., Cat. No. 12011319, Hercules, CA, USA) under the following conditions: cDNA synthesis (50 °C, 10 min) and initial inactivation (95 °C, 5 min), followed by 40 cycles of a two-step PCR comprising denaturation (95 °C, 5 s) and extension (62 °C, 20 s). A threshold cycle number ≤ 38 was considered positive. The viral RNA copy number was calculated from a standard curve showing a linear correlation between the log of the copy number and the Ct value.

The qRT-PCR analysis was also performed using a VDx^®^ BVDV qRT-PCR kit (MEDIAN diagnostics Co., Cat. No. NB-BVD-31, Chuncheon, Republic of Korea) designed to detect the 5-UTR of BVDV. The qRT-PCR conditions were as follows: cDNA synthesis (50 °C, 30 min) and initial inactivation (95 °C, 15 min), followed by 40 cycles of a two-step PCR comprising denaturation (95 °C, 10 s) and extension (60 °C, 60 s). The mean cycle threshold (Ct) values were calculated from the positive samples (i.e., those with a threshold cycle number ≤ 40).

### 2.3. Anti-CSFV E2 and Anti-CSFV E^rns^ Antibodies

CSFV E2 antibodies in porcine serum were measured using a commercially available CSFV E2 ELISA kit. Anti-CSFV E2 antibody levels were measured using the VDPro^®^ CSFV AB ELISA kit (MEDIAN diagnostic Co., Cat No. ES-CSF-01, Chuncheon, South Korea), which is an E2 protein-based ELISA. The positive control OD value should be ≥0.5, the negative control OD value should be <0.3, and the corrected positive control (CPC) value must be >0.3 (positive control OD—negative control OD). Interpretation of a positive or negative result is carried out by calculating the sample to positive (s/*p*) ratio (s/*p* = test sample OD—negative control OD/CPC OD value). For the VDPro^®^ CSFV AB ELISA kit, a sample with an s/*p* value ≥ 0.14 is considered positive for CSFV E2-specific antibodies, whereas samples with s/*p* values < 0.14 are considered negative. Pigs vaccinated with the KD26_E2LOM strain should produce anti-BVDV E^rns^ antibodies as a function of DIVA but should not produce anti-CSF E^rns^ antibodies; however, when these pigs are challenged with virulent CSFV, they become CSF E^rns^-positive. The ELISA kit used in a previous study was used to monitor CSFV E^rns^ antibody levels [15]. Anti-CSFV E^rns^ antibodies were measured using the VDPro^®^ CSF E^rns^ Ab b-ELISA (MEDIAN Diagnostic Co., Cat No. ES-CSF-05, Chuncheon, South Korea). Anti-CSFV E^rns^ positivity was based on an S/N value of ≤0.5 and anti-CSFV E^rns^ negativity on an S/N value of >0.5.

### 2.4. Serum Neutralization Assay

To perform the serum neutralization (SN) test for CSFV, serum was serially diluted 2-fold in a 96-well plate, mixed with the same amount of CSFV, and added to PK-15 cells (porcine kidney cells) [13]. The cells were cultured for 3 days (5% CO_2_, 37 °C). The cell supernatant was removed and the cells fixed for 30 min in 70% cold acetone. Next, an anti-CSFV mAb (MEDIAN diagnostic Co., Cat No. 9013, Chuncheon, Republic of Korea) was added for 1 h at 37 °C. After washing three times with PBS, the cells were stained using a VECTASTAIN^®^ ABC-HRP kit (Vector Laboratories Inc., Cat No. PK-4000, Newark, CA, USA), followed by addition of an ImmPACT DAB Peroxidase (HRP) substrate (Vector Laboratories Inc., Cat no. PK-4100, Newark, CA, USA). Staining was confirmed under a microscope. The SN antibody titer was calculated from the 2-fold dilution, at which no staining was detectable.

## 3. Results

### 3.1. Body Temperature, Leukocyte Counts, and CSFV Antigen Levels in Challenged Pigs

**Figure 1:**
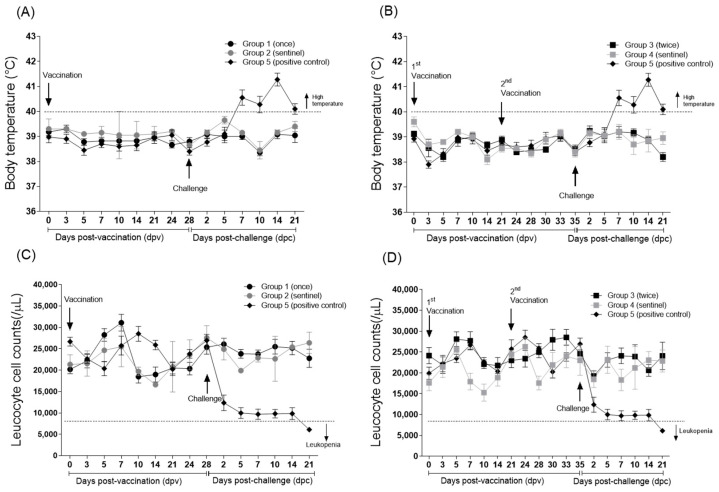
Body temperature and leukocyte counts of pigs receiving the KD26_E2 LOM vaccine, followed by challenge with virulent CSFV. Body temperature of pigs inoculated once (**A**) or twice (**B**) with the KD26_E2 LOM vaccine. Leukocyte counts of pigs inoculated once (**C**) or twice (**D**) with the KD26_E2 LOM vaccine. The error bars on the graphs represent the standard error of the mean (SEM).

The body temperature of pigs in group 1 (vaccinated once with KD26_E2 LOM strain) and in sentinel group 2 was <40 °C (Figure 1A). In addition, these pigs did not have a body temperature ≥ 40 °C on any day (Figure 1A). By contrast, positive control group 5 showed a persistently high body temperature ≥ 40 °C from 7 dpc (Figure 1A). The body temperature of pigs in group 3 (vaccinated twice with KD26_E2 LOM strain) and in sentinel group 4 was also <40 °C (Figure 1B). In addition, the white blood cell counts in groups 1 and 3, which were vaccinated with the KD26_E2 LOM strain, and in the sentinel pig groups 2 and 4 were maintained at >8000/μL before and after challenge (Figure 1C,D); however, one of the pigs in the positive control group showed leukopenia (7400/μL) from 5 dpc, and all four pigs showed leukopenia (<8000/μL) at 21 dpc (Figure 1C,D).

The VDx^®^ BVDV qRT-PCR did not detect the antigen gene of the KD26_E2 LOM strain in blood, feces, or nasal samples collected at 28 and 35 days before inoculation with virulent CSFV. In addition, the HelixDtec CSF Detection & Typing kit confirmed no virus shedding in blood, feces, or nasal samples from groups 1 to 4 at 0, 2, 5, 7, 10, 14, and 21 dpc; however, virus was detected in blood and feces from positive control group 5 at 5 dpc and in all samples (blood, nasal, and feces) continuously thereafter (Table 1). Pigs in all groups were euthanized on 21 dpc, and CSFV was detected in all organs (except brain) in the positive control group; however, no virus was detected in pigs from groups 1 to 4. The Ct values for positive control pigs were as follows: tonsil (30.3 ± 1.7), lung (32.4 ± 2.8), heart (33.8 ± 2.2), spleen (29.9 ± 2.3), liver (32.6 ± 1.5), kidney (29.9 ± 1.5), mesenteric lymph node (29.1 ± 2.3), ileum (31.9 ± 1.9), caecum (32.8 ± 2.2), and inguinal lymph node (27.9 ± 1.2).

### 3.2. Anti-CSFV and Anti-BVDV Antibody Levels

**Figure 2:**
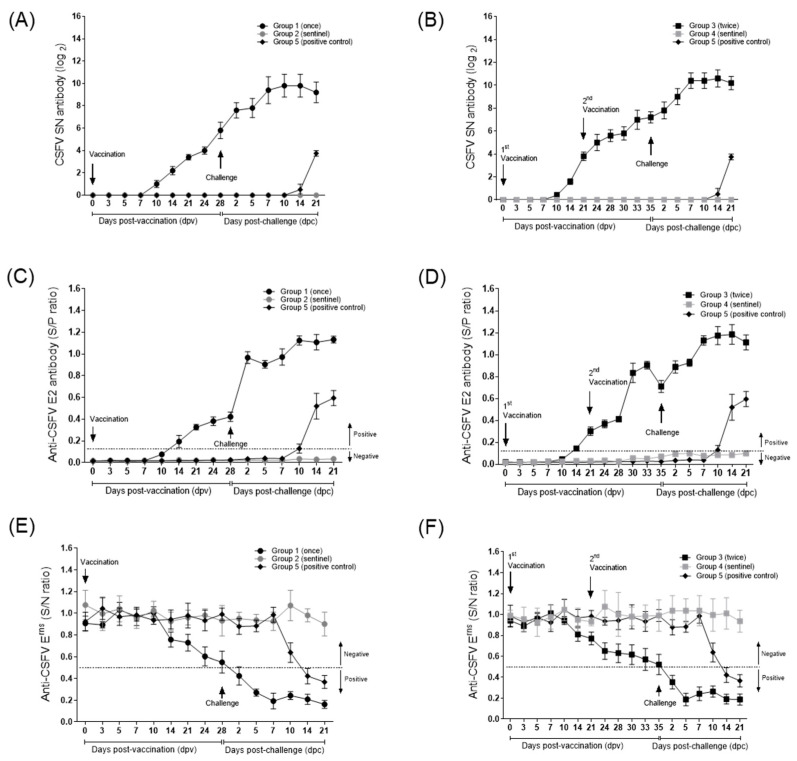
Anti-CSFV SN, E2, and E^rns^ antibody levels in pigs receiving the KD26_E2 LOM vaccine followed by challenge with virulent CSFV. CSF serum neutralizing antibody levels in pigs inoculated once (**A**) or twice (**B**) with the KD26_E2 LOM vaccine. Anti-CSFV E2 antibody levels in pigs inoculated once (**C**) or twice (**D**) with the KD26_E2 LOM vaccine. Anti-CSFV E^rns^ antibody levels in pigs inoculated once (**E**) or twice (**F**) with the KD26_E2 LOM vaccine. The error bars on the graphs represent the standard error of the mean (SEM).

In the anti-CSFV SN test, the fold changes in the mean SN values of pigs vaccinated with the KD26_E2 LOM strain were 16-fold (group 1) and 32-fold (group 3) at 24 dpv (Figure 2A,B). In addition, the fold changes in the mean SN values of pigs in group 1 were 55.7-fold at 28 dpv (0 dpc) (Figure 2A) and 147-fold at 35 dpv (0 dpc) in pigs in group 3 (Figure 2B). After challenge with virulent CSFV, the fold changes in the mean SN value of pigs in group 1 were 194-fold at 2 dpc and 891.4-fold at 10 dpc and 222.8-fold at 2 dpc and 1552-fold at 14 dpc in pigs in group 3; the results in the sentinel pigs groups (2 and 4) were negative (Figure 2A,B). After vaccination with the KD26_E2 LOM strain, the CSFV E2 ELISA revealed that the anti-CSFV E2 antibody s/p values were >0.4 (0.426 ± 0.09) at 28 dpv for pigs in group 1 and 0.83 ± 0.19 at 30 dpv for pigs in group 3 (Figure 2C,D). In addition, after challenge with virulent CSFV, the mean anti-CSFV E2 antibody levels of pigs in group 1 were maintained between 0.91 ± 0.07 (5 dpc) and 1.13 ± 0.07 (21 dpc), and those of pigs in group 3 were maintained at 0.88 ± 0.12 (2 dpc) and 1.18 ± 0.19 (14 dpc) (Figure 2C,D). However, anti-CSFV E2 antibodies were not detected in sentinel pigs (groups 2 and 4) during all observation periods (Figure 2C,D). Additionally, no anti-CSFV E2 antibodies were detected in positive control group 5 before challenge; however, the pigs became positive from 14 dpc (0.52 ± 0.23) (Figure 2C,D). The CSFV E^rns^ antibody test showed that one pig in group 1 (S/N = 0.32) was positive at 24 dpv, and two pigs (S/N = 0.29 and 0.38) were positive at 28 dpv (Figure 2E). For pigs in group 3, the CSFV E^rns^ antibody test showed one positive result at 24 dpv (S/N = 0.41), one at 28 dpv (S/N = 0.37), and two positive results at 30 (S/N = 0.39 and 0.43), 33 (S/N = 0.29 and 0.41), and 35 (S/N, 0.27 and 0.36) dpv (Figure 2F). In addition, all pigs in groups 1 and 3 became CSFV E^rns^ antibody-positive after inoculation with virulent CSFV (Figure 2E,F).

## 4. Discussion

Development of a next-generation CSFV live marker vaccine should focus on the DIVA concept, which enables protection against virulent CSFV and discrimination of antibodies generated by the vaccine or live virus. There are two commercialized chimeric *pestiviruses.* CP7_E2alf, Flc-LOM-BE^rns^ is based on infectious cDNA clones of CSFV or BVDV [16,17,18,19]. The CP7_E2alf vaccine was constructed using an infectious cDNA clone of cytopathogenic BVDV (strain CP7), in which the E2 gene was replaced with that from CSFV (strain Alfort/187) [8,16,20]. Pigs vaccinated with CP7_E2Alf were fully protected against challenge with the same highly virulent CSFV strain (Koslov strain) after 1 week, with partial protection against an intermediate virulent strain reported as early as 2 dpv [21,22]. Another chimeric *pestivirus* (Flc-LOM-BE^rns^) is based on a live attenuated CSFV vaccine (strain LOM), in which the complete E^rns^ and signal peptide-encoding sequences have been replaced with the corresponding BVDV sequences [15]. Pigs vaccinated with Flc-LOM-BE^rns^ were fully protected against challenge with virulent CSFV after 1 week, and there were no problems with respect to vertical protection in pregnant sows [15]. Recent studies generated chimeric virus candidates based on the hypothesis that genetically different *pestiviruses* can generate replicative chimeric viruses with improved DIVA capacity and with a significantly reduced risk of inducing cross-reactive antibodies [9,10]. Three chimeric *pestivirus* vaccine candidates (Ra, Pro, and RaPro) provided robust protection, with no clinical signs of CSF at day 28 postvaccination. More recently, novel chimeric viruses were constructed for use as CSF live marker vaccine candidates; these vaccines were based on the backbone of the Japanese GPE^−^ vaccine strain, although the glycoprotein E^rns^ was replaced with that from PAPeV or PhoPeV [11,12]. Pigs immunized with these chimeric viruses, called vGPE^−^/PAPeV E^rns^ or vGPE^−^/PhoPeV E^rns^, respectively, showed no clinical signs or systemic virus replication at 7 dpc with a moderately virulent CSFV strain [12]. In this study, specific clinical symptoms of CSF (i.e., high fever and leukopenia) were not observed on day 28 in pigs vaccinated with KD26_E2 LOM or on day 35 in pigs that received a second dose of the vaccine. By contrast, positive control pigs showed high fever and leukopenia. This suggests that if the pigs mount a sufficiently strong immune response to the KD26_E2 LOM vaccine, they will be protected against infection by virulent CSFV. In addition, our data suggest that transmission of CSFV from pig to pig can be prevented because the pigs inoculated with the KD26_E2 LOM vaccine did not shed virus after challenge, and neither CSFV antigens nor anti-CSFV antibodies were detected in sentinel pigs.

Recent studies that developed chimeric viruses focused on the DIVA concept, which allows discrimination of antibodies generated by a vaccine from those generated by natural infection; these studies attempted to create replicating chimeric *pestiviruses* with improved marker characteristics but with a lower risk of generating cross-reactive antibodies to genetically distinct *pestiviruses*. Data from E^rns^-based differential ELISA suggest that the previously developed CP7_E2alf vaccine, which is based on infectious cDNA clones of BVDV, generates cross-reactive antibodies in the sera of BDV- and BVDV-infected animals [23,24]. In the case of another chimeric *pestivirus* (Flc-LOM-BE^rns^), experiments with large numbers of pigs suggested possible cross-reactivity, although it was not reported in the limited number of pigs used in that study [15]. The immunity induced by the three chimeric *pestivirus* vaccine candidates mentioned above (Ra, Pro, and RaPro) showed delayed immunity when compared with that induced by the CP7_E2alf vaccine [10], but DIVA was demonstrated to be feasible, as a CSFV E^rns^ antibody-based DIVA assay detected no cross-reactivity in sera from vaccinated pigs [9]. Therefore, development of chimeric *pestivirus* vaccines containing the E^rns^ sequences of distantly related *pestiviruses* is a promising strategy for establishing improved serological negative markers for CSF DIVA vaccines [9]. The anti-CSFV E2 and anti-CSFV E^rns^ antibodies detected in the pigs inoculated with the KD26_E2 LOM vaccine in the present study were distinguishable from each other for up to 21 dpv; however, all pigs generated anti-CSFV E2 antibodies between 24 and 35 dpv, and some developed anti-CSFV E^rns^ antibodies. In group 1, which was inoculated once with the KD26_E2 LOM vaccine, anti-CSFV E^rns^ antibodies were observed in 20% (1/5) of pigs at 24 dpv and 40% (2/5) at 28 dpv, and in group 3, which was inoculated twice, antibodies were observed in 20% (1/5) at 24 dpv, 40% (2/5) at 30 dpv, and 40% (2/5) at 35 dpv. Anti-CSFV E^rns^ antibodies were similar in pigs regardless of whether KD26_E2 LOM vaccines were applied once or twice, which does not allow a differential diagnosis. This is probably due to the high amino acid homology (73.1%) between the E^rns^ genes of BVDV KD26 strain E^rns^ and CSFV (LOM strain), which may lead to high E^rns^ antibody cross-reactivity [13]. Therefore, further experiments using a greater number of pig samples, along with an improved CSFV E^rns^ ELISA, are needed to allow a better understanding of the differential detection potential of the KD26_E2 LOM vaccine. The live attenuated vaccine (strain LOM) and the chimeric *pestivirus* (strain Flc-LOM-BE^rns^) provided complete protection (32-fold) and 50% protection (16-fold) after active immunization [25]. In addition, maternal antibodies induced by the Flc-LOM-BE^rns^ vaccine provided half-protection at 32-fold and complete protection at 64-fold [25]. Pigs immunized with chimeric viruses vGPE^−^ /PAPeV E^rns^ or vGPE^−^ /PhoPeV E^rns^ demonstrated early protective efficacy against challenge 7 days later with a moderately virulent CSFV [12]; however, pigs inoculated with the KD26_E2 LOM vaccine formed neutralizing antibodies (16-fold or 32-fold) at 24 dpv, making it difficult to justify its use as an early protective vaccine candidate.

## 5. Conclusions

In conclusion, the KD26_E2 LOM vaccine showed protective efficacy against a moderately virulent CSFV strain and protected sentinel pigs from CSFV transmission; however, it is difficult to justify its use as an early protective vaccine against CSFV. Additional experiments are required to test DIVA function in more detail and facilitate differential diagnosis.

## Figures and Tables

**Table 1:** Detection of CSFV in blood, nasal, and fecal samples days after CSFV inoculation.

Group	Vaccine (no. of Injections)	No. Pigs	Days after CSFV Inoculation						
			0	2	5	7	10	14	21
1	KD26_E2 LOM strain (one)	5	0/0/0	0/0/0	0/0/0	0/0/0	0/0/0	0/0/0	0/0/0
2	Sentinel with group 1	2	0/0/0	0/0/0	0/0/0	0/0/0	0/0/0	0/0/0	0/0/0
3	KD26_E2 LOM strain (two)	5	0/0/0	0/0/0	0/0/0	0/0/0	0/0/0	0/0/0	0/0/0
4	Sentinel with group 3	2	0/0/0	0/0/0	0/0/0	0/0/0	0/0/0	0/0/0	0/0/0
5	Positive control	4	0/0/0	0/0/0	4/0/4 *	4/4/4	4/4/4	4/4/4	4/4/4

*: Number of positive blood/nasal/fecal samples.

## Data Availability

Data are available from the corresponding author upon reasonable request.
